# Acute Retention of Urine—III

**Published:** 1908-07-18

**Authors:** 


### Surgery.

### ACUTE RETENTION OF URINE—III.

### OPERATIVE TREATMENT.

Internal urethrotomy is not an operation which is indicated in acute retention of urine, for it is possible only if the stricture admits a No. 4 or 5 English bougie. It is particularly useful in patients who are found to suffer from catheter fever on the passage of an instrument or in those who have a resilient stricture—that is to say, one which contracts down again to its original size immediately after the passage of an instrument. The same objection holds in the case of Syme's external urethrotomy, in which a shouldered staff is passed through the stricture and an incision made along it. In either case the stricture must be dilated sufficiently to admit the passage of an instrument of small calibre, and if this can be done at all the urine can be withdrawn and the stricture treated by interrupted dilatation in the ordinary way.

Acute retention which resists all other forms of treatment demands, therefore, either Cock's or Wheelhouse's operation. But before proceeding to either of these extremes the surgeon must be completely satisfied that every instrumental method of overcoming the stricture has been tried and failed. Of the two Wheelhouse's operation is preferable, and will alone be described in detail. In Cock's operation a median perineal section is performed, and an opening is made into the urethra posterior to the stricture. This is not so difficult as might be supposed, since the proximal portion of the urethra in these cases is generally so hypertrophied and dilated as to be easily recognisable. If this were not the case it would be almost impossible to find it, unless a guide had been previously passed down to the stricture. The stricture itself is cut through from behind forwards, and the rest of the treatment does not differ from that of Wheelhouse's operation.

In this a staff with a hooked end is passed down the urethra as far as the stricture. The patient is placed in the lithotomy position and an incision is made about one and a half or two inches long in the middle line; its centre should correspond to a point situated half-way between the anus and the posterior edge of the scrotum. The incision is gradually deepened and the hooked end of the staff carefully felt for. During this part of the operation the onus of responsibility falls mainly upon the assistant, for it is his duty to see that the patient lies flat upon his back, and to hold the staff exactly in the middle line. Lack of care about either of these precautions may engender difficulty; for the surgeon will imagine himself to be going in the middle line, while he is in reality groping all the time to one or other side of the urethra. It is astonishing how easy it is to miss the urethra altogether, in spite of the fact that it contains a staff to guide one.

When the urethra is found an incision is made through its inferior wall on to the hooked end of the staff, which is then pushed through the aperture so made and turned so that the hook catches in the angle of the incision in the urethra. By this means the urethra is gently held on the stretch, and the stricture is incised from before backwards. It sometimes happens that as soon as this is done urine comes away from the bladder of its own accord, but as often as not the muscles of the bladder have become over-distended and atonic, so that they are unable to expel the urine. The sphincter must, therefore, be dilated to help them. A special instrument has been devised with this object. It is known as Pridgen Teale's probe-pointed gorget. It consists of a tapering director, whose point is guarded by a small metal sphere. It will be found, however, that an ordinary surgical director will answer the purpose equally well. Whichever instrument is used it is passed into the bladder, and the moment it passes through the neck there is a gush of urine, and the bladder is quickly emptied.

The next step is to pass a gum-elastic catheter, preferably a No. 10 or 12, into the bladder per urethram. This must be done while the patient is still in the lithotomy position and before the wound is closed, because the point has to be directed with the finger past the site of the stricture. There is some difference of opinion as to whether the wound should be sewn up or left open. As a matter of fact, it makes but little difference whichever alternative is chosen. If the wound is sewn up, primary union hardly ever occurs, because some urine is bound to leak through; while if it is left open, the natural position of the parts when the legs are extended again tends to keep the edges in apposition, so that in any case a fairly good scar results.

Another small but important point is the method of fixing in the catheter. This is mostly simply done by making a clove hitch with some tape, tightening it round the protruding end of the catheter, and fixing the loose ends to the penis with strapping. In spite of this the catheter sometimes comes out during the night, and if this happens a more effectual, though necessarily more painful, method is to pass a stitch through the catheter and through the prepuce and tie it in position. It is important to see that the catheter is firmly fixed in, because if it comes out in the first twenty-four hours it may be found impossible to re-introduce it without opening up the wound and practically doing the operation over again.

In the ordinary course of events the catheter should be allowed to remain *in situ* as long as possible, being merely turned round once daily. After a few days, however, a discharge will be seen oozing from between the catheter and the lips of the meatus. The time has then come to change it. On the first occasion it is well to give an anæsthetic, so that the patient lies still and does not struggle. Afterwards, when a false urethra is more or less formed, it is a simpler matter. Finally it must be remembered that the patient who has had an external urethrotomy performed should have an instrument passed about once a month, because the urethra formed by the operation shows as great a tendency to contract as any other fibrous tissue, and this is an inconvenience which he will suffer to the end of his days.

